# Supplementary material for: Directed evolution reveals the mechanism of HitRS signaling transduction in Bacillus anthracis
Source: PLoS Pathog. 2020 Dec 23;16(12):e1009148. doi: 10.1371/journal.ppat.1009148 (PMC7790381; doi:10.1371/journal.ppat.1009148)
Supplement: S1 Table — (PDF) [file ppat.1009148.s001.pdf]

S1 Table. Bacterial strains and plasmids used in this study

Species	Genotype	Description	Reference
B. anthracis strain Sterne	WT	Wildtype laboratory stock	Lab stock
B. anthracis strain Sterne	P_{hit}*ermC*	*hit* promoter fused to erythromycin resistance gene *ermC* and inserted into the *BAS3009* pseudogene locus (*bas3009::hit-ermC*)	This study
B. anthracis strain Sterne	2(*relE*)	*hit* promoter fused to *relE* and inserted into the *BAS3009* and *BAS4599* pseudogene loci (*bas3009::hit-relE bas4599::hit-relE*)	This study
B. anthracis strain Sterne	2(*relE* + *hitRS*)	*hit* promoter fused to *relE* and inserted into the *BAS3009* and *BAS4599* pseudogene loci; second copy of *hitRS* inserted into the *BAS4927* pseudogene locus (*bas3009::hit-relE bas4599::hit-relE bas4927::hitRS*)	This study
B. anthracis strain Sterne	*hitR:M58I*	Chromosomal *hitRS* point mutant	This study
B. anthracis strain Sterne	*hitR:R192A*	Chromosomal *hitRS* point mutant	This study
B. anthracis strain Sterne	*hitS:T118I*	Chromosomal *hitRS* point mutant	This study
B. anthracis strain Sterne	*hitS:S141L*	Chromosomal *hitRS* point mutant	This study
B. anthracis strain Sterne	*hitS:N248S*	Chromosomal *hitRS* point mutant	This study
E. coli strain DH5α	WT	Wildtype laboratory stock for cloning	Lab stock
E. coli strain K1077	WT	Wildtype laboratory stock for cloning	Lab stock

Plasmid	Description	Reference
pLM4	Allelic exchange vector for *B. anthracis*	Lab stock
pCR2.1.*ermC*	Erythromycin resistance gene from *Staphylococcus aureus* cloned into plasmid pCR2.1	This study
pLM4-*3009*	Vector to integrate genes within *BAS3009*	This study
pLM4-*4599*	Vector to integrate genes within *BAS4599*	This study
pLM4-*4927*	Vector to integrate genes within *BAS4927*	This study
pLM4-*3009::*P_{hit}*ermC*	Vector to integrate *hit-ermC* fusion within *BAS3009*	This study
pLM4-*3009::*P_{hit}*relE*	Vector to integrate *hit-relE* fusion within *BAS3009*	This study
pLM4-*4599::*P_{hit}*relE*	Vector to integrate *hit-relE* fusion within *BAS4599*	This study
pLM4-*4927::hitRS*	Vector to integrate *hitRS* within *BAS4927*	This study
pLM4-*hitRS*	Template for site-directed mutagenesis to generate *hitRS* point mutants in chromosomal *hitRS*	This study

pLM4-*hitR:M58I*	Plasmid for *hitRS* point mutant in chromosomal *hitRS*	This study
pLM4-*hitR:R192A*	Plasmid for *hitRS* point mutant in chromosomal *hitRS*	This study
pLM4-*hitS:T118I*	Plasmid for *hitRS* point mutant in chromosomal *hitRS*	This study
pLM4-*hitS:S141L*	Plasmid for *hitRS* point mutant in chromosomal *hitRS*	This study
pLM4-*hitS:N248S*	Plasmid for *hitRS* point mutant in chromosomal *hitRS*	This study
