# Supplementary material for: Directed evolution reveals the mechanism of HitRS signaling transduction in Bacillus anthracis
Source: PLoS Pathog. 2020 Dec 23;16(12):e1009148. doi: 10.1371/journal.ppat.1009148 (PMC7790381; doi:10.1371/journal.ppat.1009148)
Supplement: S2 Table — (PDF) [file ppat.1009148.s002.pdf]

S2 Table: Oligonucleotide primers used for this study

Primer name	Sequence	Use
*3009*_XmaI_fwd	GCATGACCCGGGTTGCAATAGGAATTGTG	Chromosomal insertion
*3009*_SOE-L	GCTAGCGCATGGTACCTTACTCTCCAAAATATCCATAAGC	Chromosomal insertion
*3009*_SOE-R	GTAAGGTACCATGCGCTAGCCCTAGTTACCAATTTACCGAAC	Chromosomal insertion
*3009*_SacI_rev	GCATGAGAGCTCAACTCTCCACAAATAAGCAGC	Chromosomal insertion
*4599*_XmaI_fwd	GCATGACCCGGGGATGATTTAATTCGTGATACAC	Chromosomal insertion
*4599*_SOE-L	GCTAGCGCTACTGGTACCTAGCAGTTTGTAAATACTCTTC	Chromosomal insertion
*4599*_SOE-R	GGTACCAGTAGCGCTAGCAAAAAATAGGTGAAGCAATTGTAG	Chromosomal insertion
*4599*_SacI_rev	GCATGAGAGCTCCCTCCAAAATAGAGAGCGG	Chromosomal insertion
*4927*_XmaI_fwd	GCATGACCCGGGAACCGTAACTCTACATCATGG	Chromosomal insertion
*4927*_SOE-L	GCTAGCGCATGCGGTACCCCATACTCGAACTGCATACC	Chromosomal insertion
*4927*_SOE-R	GGTACCGCATGCGCTAGCTGTAGTGAAAGTATTCCACGC	Chromosomal insertion
*4927*_SacI_rev	GCATGAGAGCTCTTCAACAGCTGTTCCGATTAC	Chromosomal insertion
P_{hit}*ermC*_NheI_fwd	GCATGAGCTAGCCTCTTTTTTGAAGGCACGACG	Chromosomal insertion
P_{hit}*ermC*_SOE_L	CTCGTTCATTCATGTTCATCTCCTCGTG	Chromosomal insertion
P_{hit}*ermC*_SOE_R	GAGATGAACATGAATGAACGAGAAAAATATAAAACAC	Chromosomal insertion
P_{hit}*ermC*_KpnI_rev	GCATGAGGTACCTTACTTATTAAATAATTTATAGCTATTG	Chromosomal insertion
P_{hit}*relE*_NheI_fwd	GCATGAGCTAGCATGCATCTGCAGCTCTTTTTTGAAGGCACGACG	Chromosomal insertion
P_{hit}*relE*_SOE_L	GAAAATACGCCATTCATGTTCATCTCCTCGTG	Chromosomal insertion
P_{hit}*relE*_SOE_R	GATGAACATGAATGGCGTATTTTCTGGATTTTG	Chromosomal insertion
P_{hit}*relE*_KpnI_rev	GCATGAGGTACCTCAGAGAATGCGTTTGACCG	Chromosomal insertion
*hitRS*_KpnI_fwd	GCATGACCCGGGGGTACCTGACATGTATCGTGAATTTGC	Chromosomal insertion
*hitRS*_NheI_rev	GCATGAGAGCTCGCTAGCCAGTATAGCTGCAGTAATGG	Chromosomal insertion
*hitRS*_XmaI_fwd	GCATGACCCGGGAGTAAGTCCGAGTCCGTCG	Chromosomal insertion
*hitRS*_SacI_rev	GCATGAGAGCTCATCAGCAGTTGCAATAACAGC	Chromosomal insertion
*hitR:M58I*_fwd	TCTTGATATCattATGCCGAATATG	Site-directed mutagenesis
*hitR:M58I*_rev	ATAACCATATCAACTTTCACTTG	Site-directed mutagenesis
*hitR:R192A*_fwd	AGGGAATGAAgcaACACTAGACGTTCATATAAATC	Site-directed mutagenesis

*hitR:R192A*_rev	TCAAAATCATATCCCCATAC	Site-directed mutagenesis
*hitS:T118I*_fwd	AAACGATATGattGATGAACTGAATGCG	Site-directed mutagenesis
*hitS:T118I*_rev	ATACTTTTTACGAGCACAC	Site-directed mutagenesis
*hitS:S141L*_fwd	TGAAATACAGcttCCATTAACTTCTATAAAAGG	Site-directed mutagenesis
*hitS:S141L*_rev	TGAGAAACATTCGAAACAAATTC	Site-directed mutagenesis
*hitS:N248S*_fwd	AGTTTGGATTagtTTAATTCATAATAGTATTAAATTTAC	Site-directed mutagenesis
*hitS:N248S*_rev	TGACTCATACTTTCTTGG	Site-directed mutagenesis
