# Supplementary material for: Directed evolution reveals the mechanism of HitRS signaling transduction in Bacillus anthracis
Source: PLoS Pathog. 2020 Dec 23;16(12):e1009148. doi: 10.1371/journal.ppat.1009148 (PMC7790381; doi:10.1371/journal.ppat.1009148)
Supplement: S3 Table — (PDF) [file ppat.1009148.s003.pdf]

S3 Table: Point mutations within HitRS affect the signaling pathway in various manners

Protein	Mutation	Domain	Protein stability	Dimerization	Kinase activity	Phosphotransfer activity	Phosphatase activity	DNA-binding affinity	Overall effect
HitS	M117V	HAMP	--	N	abolished	NA	--	NA	OFF
	T118I		N	N	N	+	N	NA	ON
	S136F	DHp	N	N	--	+	N	NA	ON
	H137A		N	N	abolished	NA	N	NA	OFF
	S141L		N	N	N	+	--	NA	ON
	F149L		N	N	--	+	--	NA	ON
	D227V	CA	N	N	++	+	N	NA	ON
	N248S		-	N	abolished	NA	--	NA	OFF
	V274A		-	N	-	+	N	NA	ON
	A300E		N	N	+	+	N	NA	ON
	R306S		N	N	+	+	--	NA	ON
	G309R		N	N	--	+	N	NA	ON
HitR	D56N	Receiver	N	N	NA	abolished	NA	abolished	OFF
	M58I		N	N	NA	+	NA	++	ON
	F95S		N	N	NA	-	NA	abolished	OFF
	P106S		-	N	NA	--	NA	---	OFF
	P155L	DNA-binding	N	N	NA	-	NA	abolished	OFF
	K168A		N	+	NA	+	NA	+++	ON
	R192C		N	+	NA	-	NA	abolished	OFF
	E203A		N	-	NA	N	NA	+++	ON
	Y222D		--	N	NA	-	NA	---	OFF

N denotes no significant effects; "-" denotes negative effects; "+" denotes positive effects; NA, not applicable; ON, constitutively activation; OFF, inactivation
