# Supplementary material for: Directed evolution reveals the mechanism of HitRS signaling transduction in Bacillus anthracis
Source: PLoS Pathog. 2020 Dec 23;16(12):e1009148. doi: 10.1371/journal.ppat.1009148 (PMC7790381; doi:10.1371/journal.ppat.1009148)
Supplement: S1 Fig — (A) Multiple HK sequence were aligned to display the conserved motifs in the kinase core domains of HKs. The two helices of DHp domain are indicated and conserved hydrophobic residues in helix 2 are shaded in gray. The H, N, D (or G1), F, G2, and G3-boxes are shown in boxes. The RR-binding motif is underlined, the hydrophobic residues in the HAMP2 helix are highlighted in gray, and the hydrophobic residues that form the Gripper fingers are highlighted in cyan. The residues between F and G2-boxes form a loop structure known as ATP-lid that holds the ATP molecule. The ATP-lid, Gripper helix, N, D, and G-boxes are involved in ATP-binding and phosphotransfer. The residues identified from genetic selections are highlighted in either orange or blue to specify either ON or OFF mutations, respectively. The sequences are the following: HitS, B. anthracis; HK853, Thermotoga maritima; PhoQ, E. coli; EnvZ, E. coli; WalK, Staphylococcus aureus; PhoR, E. coli; ArcB, E. coli. (B) The four-helix bundle of DHp domain is divided into three segments based on sequence analysis [15] and all residues highlighted in orange or blue were identified by genetic selections. The homology modelling was based on HK853 (PDB ID: 4JAU). (C) All features in the CA domain are highlighted in the model structure, which was based on HK853 (PDB ID: 4RH8). OFF mutations are shown in blue while ON mutations are shown in orange. (PDF) [file ppat.1009148.s004.pdf]

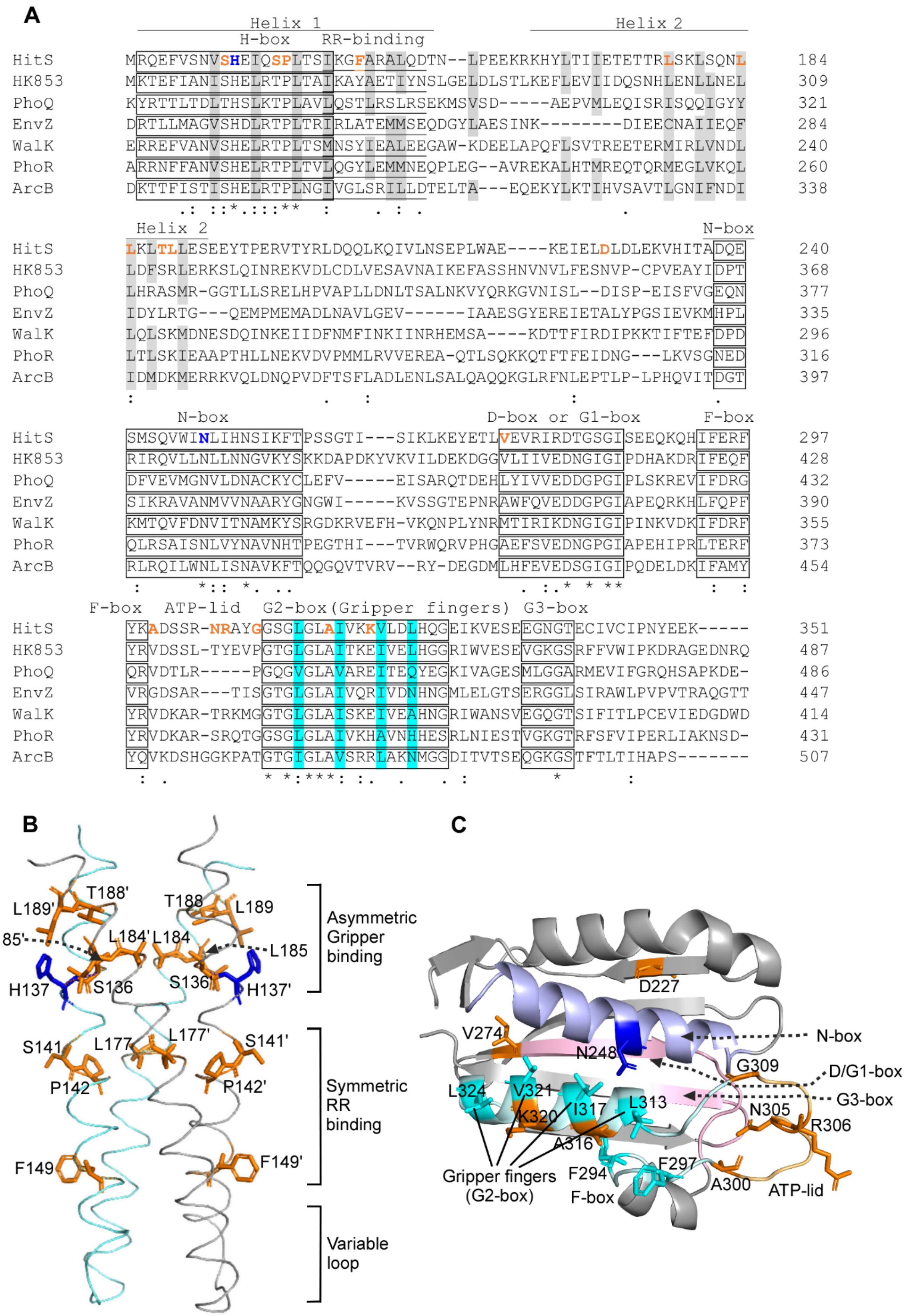
A
Helix 1
Helix 2
H-box
RR-binding
HitS MRQEFVSNVSHEIQSPLTSIKGFARALQDTN--LPEEKRKHYLTIIETETTRLSKLSQNL 184
HK853 MKTEFIANISHELRTPLTAIKAYAETIYNSLGELDLSTLKEFLEVIIDQSNHLENLLNEL 309
PhoQ KYRTTLTDLTHSLKTPLAVLQSTLRSLRSEKMSVSD-----AEPVMLEQISRISQQIGYY 321
EnvZ DRTLLMAGVSHDLRTPLTRIRLATEMMSEQDGYLAESINK--------DIEECNAIIEQF 284
WalK ERREFVANVSHELRTPLTSMNSYIEALEEGAW-KDEELAPQFLSVTREETERMIRLVNDL 240
PhoR ARRNFFANVSHELRTPLTVLQGYLEMMNEQPLEG--AVREKALHTMREQTQRMEGLVKQL 260
ArcB DKTTFISTISHELRTPLNGIVGLSRILLDTELTA---EQEKYLKTIHVSAVTLGNIFNDI 338
.: ::*.:::** : . : . .
Helix 2
N-box
HitS LKLTTLLESEEYTPERVTYRLDQQLKQIVLNSEPLWAE----KEIELDLDLEKVHITADQE 240
HK853 LDFSRLERKSLQINREKVDLCDLVESAVNAIKEFASSHNVNVLFESNVP-CPVEAYIDPT 368
PhoQ LHRASMR-GGTLLSRELHPVAPLLDNLTSALNKVYQRKGVNISL--DISP-EISFVGEQN 377
EnvZ IDYLRTG---QEMPMEMADLNAVLGEV------IAAESGYEREIETALYPGSIEVKMHPL 335
WalK LQLSKMDNESDQINKEIIDFNMFINKIINRHEMSA----KDTTFIRDIPKKTIFTEFDPD 296
PhoR LTLSKIEAAPTHLLNEKVDVPMMLRVVEREA-QTLSQKKQTFTFEIDNG---LKVSGNED 316
ArcB IDMDKMERRKVQLDNQPVDFTSFLADLENLSALQAQQKGLRFNLEPTLP-LPHQVITDGT 397
: . : : .
N-box
D-box or G1-box
F-box
HitS SMSQVWINLIHNSIKFTPSSGTI---SIKLKEYETLVEVRIRDTGSGISEEQKQHIFERF 297
HK853 RIRQVLLNLLNNGVKYSKKDAPDKYVKVILDEKDGGVLIIVEDNGIGIPDHAKDRIFEQF 428
PhoQ DFVEVMGNVLDNACKYCLEFV-----EISARQTDEHLYIVVEDDGPGIPLSKREVIFDRG 432
EnvZ SIKRAVANMVVNAARYGNGWI-----KVSSGTEPNRAWFQVEDDGPGIAPEQRKHLFQPF 390
WalK KMTQVFDNVITNAMKYSRGDKRVEFH-VKQNPLYNRMTIRIKDNGIGIPINKVDKIFDRF 355
PhoR QLRSAISNLVYNAVNHTPEGTHI---TVRWQRVPHGAEFSVEDNGPGIAPEHIPRLTERF 373
ArcB RLRQILWNLISNAVKFTQQGQVTVRV--RY-DEGDMLHFEVEDSGIGIPQDELDKIFAMY 454
: *:: *. .. . :.* * ** : :
F-box
ATP-lid
G2-box(Gripper fingers)
G3-box
HitS YKADSSR-NRAYGGSGLGLAIVKKVLDLHQGEIKVESEEGNGTECIVCIPNYEEK----- 351
HK853 YRVDSSL-TYEVPGTGLGLAITKEIVELHGGRIWVESEVGKGSRFFVWIPKDRAGEDNRQ 487
PhoQ QRVDTLR-----PGQGVGLAVAREITEQYEGKIVAGESMLGGARMEVIFGRQHSAPKDE- 486
EnvZ VRGDSAR---TISGTGLGLAIVQRIVDNHNGMLELGTSERGGLSIRAWLPVPVTRAQGTT 447
WalK YRVDKAR-TRKMGGTGLGLAISKEIVEAHNGRIWANSVEGQGTSIFITLPCEVIEDGDWD 414
PhoR YRVDKAR-SRQTGGSGLGLAIVKHAVNHHESRLNIESTVGKGTRFSFVIPERLIAKNSD- 431
ArcB YQVKDSHGGKPATGTGIGLAVSRRLAKNMGGDITVTSEQGKGSTFTLTIHAPS------- 507
: . * *:***: :. . : * :
B
L189'
T188'
T188
L189
L185'
L184'
L184
L185
H137
S136
S136'
H137'
S141
L177
L177'
S141'
P142
P142'
F149
F149'
Asymmetric
Gripper
binding
Symmetric
RR
binding
Variable
loop
C
D227
V274
N248
N-box
G309
D/G1-box
G3-box
L324
V321
K320
I317
L313
N305
R306
A316
F294
F297
A300
ATP-lid
Gripper fingers
(G2-box)
F-box

S1 Fig. Conserved motifs in the kinase core domains

(A) Multiple HK sequence were aligned to display the conserved motifs in the kinase core domains of HKs. The two helices of DHp domain are indicated and conserved hydrophobic residues in helix 2 are shaded in gray. The H, N, D (or G1), F, G2, and G3-boxes are shown in boxes. The RR-binding motif is underlined, the hydrophobic residues in the HAMP2 helix are highlighted in gray, and the hydrophobic residues that form the Gripper fingers are highlighted in cyan. The residues between F and G2-boxes form a loop structure known as ATP-lid that holds the ATP molecule. The ATP-lid, Gripper helix, N, D, and G-boxes are involved in ATP-binding and phosphotransfer. The residues identified from genetic selections are highlighted in either orange or blue to specify either ON or OFF mutations, respectively. The sequences are the following: HitS, *B. anthracis*; HK853, *Thermotoga maritima*; PhoQ, *E. coli*; EnvZ, *E. coli*; WalK, *Staphylococcus aureus*; PhoR, *E. coli*; ArcB, *E. coli*. (B) The four-helix bundle of DHp domain is divided into three segments based on sequence analysis (15) and all residues highlighted in orange or blue were identified by genetic selections. The homology modelling was based on HK853 (PDB ID: 4JAU). (C) All features in the CA domain are highlighted in the model structure, which was based on HK853 (PDB ID: 4RH8). OFF mutations are shown in blue while ON mutations are shown in orange.
