# Supplementary material for: Directed evolution reveals the mechanism of HitRS signaling transduction in Bacillus anthracis
Source: PLoS Pathog. 2020 Dec 23;16(12):e1009148. doi: 10.1371/journal.ppat.1009148 (PMC7790381; doi:10.1371/journal.ppat.1009148)
Supplement: S2 Fig — (A) Sequence alignment of the receiver domains. The sequences are the following: HitR; BfmR, Acinetobacter baumannii; PhoP, Bacillus subtilis; YycF, Bacillus subtilis; PhoB, E. coli; and RstA, A. baumannii. (B) Sequence alignment of the DNA-binding domains. The sequences are the following: HitR; PrrA, Mycobacterium tuberculosis; PhoB, E. coli; SaeR, Staphylococcus aureus; VicR, Streptococcus mutans; RegX3, Mycobacterium tuberculosis; YycF, Bacillus subtilis. The α-helices and β-sheets are underlined. The homology models of the receiver (C) and DNA-binding domain (D) of HitR were generated based on M. tuberculosis RegX3 (PDB ID: 2OQR), which is in an active dimer form. Residues identified from genetic selections are highlighted in either orange or blue to specify either ON or OFF mutations, respectively. (PDF) [file ppat.1009148.s005.pdf]

**A. Receiver domain**

```
           ß1        a1          ß2        a2          ß3       a3
HitR      NILIVDDDPHIRELVSVFLECEGFQTYEAIDGLDALQKINQVKVDMVILDIMMPNMDGFD
BfmR      KILIVEDDERLARLTQEYLIRNGLEVGVETDGNRAIRRIISEQPDLVVLDVMLPGADGLT
PhoP      -ILVVDDEESIVTLLQYNLERSGYDVITASDGEEALKKAETEKPDLIVLDVMLPKLDGIE
YycF      -ILVVDDEKPIADILEFNLRKEGYEVHCAHDGNEAVEMVEELQPDLILLDIMLPNKDGVE
PhoB      RILVVEDEAPIREMVCFVLEQNGFQPVEAEDYDSAVNQLNEPWPDLILLDWMLPGGSGIQ
RstA      KILIVEDDERLARLTQEYLIRNGLEVGVETDGNRAIRRIISEQPDLVVLDVMLPGADGLT
           **:*:*:  :  :    *  .* :     *   *:.       *:::** *:*  .*.

             a3         ß4          a4     ß5          a5
HitR      VCFELRK-Y--YDIPILILTAKGETSQKVKGFHLGTDDYLVKPFDPIELVVRVKALLKR
BfmR      VCREVRP-H--YHQPILMLTARTEDMDQVLGLEMGADDYVAKPVQPRVLLARIRALLRR
PhoP      VCKQLRQQK--LMFPILMLTAKDEEFDKVLGLELGADDYMTKPFSPREVNARVKAILRR
YycF      VCREVRK-K--YDMPIIMLTAKDSEIDKVIGLEIGADDYVTKPFSTRELLARVKANLRR
PhoB      FIKHLKRESMTRDIPVVMLTARGEEEDRVRGLETGADDYITKPFSPKELVARIKAVMRR
RstA      VCREVRP-H--YHQPILMLTARTEDMDQVLGLEMGADDYVAKPVQPRVLLARIRALLRR
          .  .::        *:::***: .  ::* *:. *:***:.**..   : .*::* ::*
```

**B. DNA binding domain**

```
              ß8           a6            a7             a8
HitR      NRKTFEVTIGEQTVTLPLKEFELLFTLGSKAGRTCSREQLIEDVWGYDFEGNERTLDVHI
PrrA      DIPGRRARVNGVDVDLTKREFDLLAVLAEHKTAVLSRAQLLELVWGYDFAADTNVVDVFI
PhoB      DPTSHRVMAGEEPLEMGPTEFKLLHFFMTHPERVYSREQLLNHVWGTNVYVEDRTVDVHI
SaeR      INLSKVVTVNGHEVPMRIKEFELLWYLASRENEVISKSELLEKVWGYDYYEDANTVNVHI
VicR      LPDAFVAKKRGTEVELTHREFELLHHLATHTGQVMTREHLLETVWGYDYFGDVRTVDVTV
RegX3     --ERHVVSVNGDTITLPLKEFDLLEYLMRNSGRVLTRGQLIDRVWGADYVGDTKTLDVHV
YycF      FPDAYVVSKRDETIELTHREFELLHYLAKHIGQVMTREHLLQTVWGYDYFGDVRTVDVTV
                .      : :   **.**  :  .   . :: .*:: *** :   : ..::* :

            a8          ß9   ß10
HitR      NRLREKFQE-EKSKFSIKTIRGLGYRLEVSK
PrrA      GYLRRKLEA-GGGPRLLHTVRGVGFVLRMQ-
PhoB      RRLRKALEP-GGHDRMVQTVRGTGYRFSTR-
SaeR      HRIREKLEKESFTTYTITTVWGLGYKFERS-
VicR      RRLREKIEDTPSRPEYILTRRGVGYYMKS--
RegX3     KRLRSKIEADPANPVHLVTVRGLGYKLE---
YycF      RRLREKIEDNPSHPNWIVTRRGVGYYLRNP-
            :*  ::        : *  * *: :
```

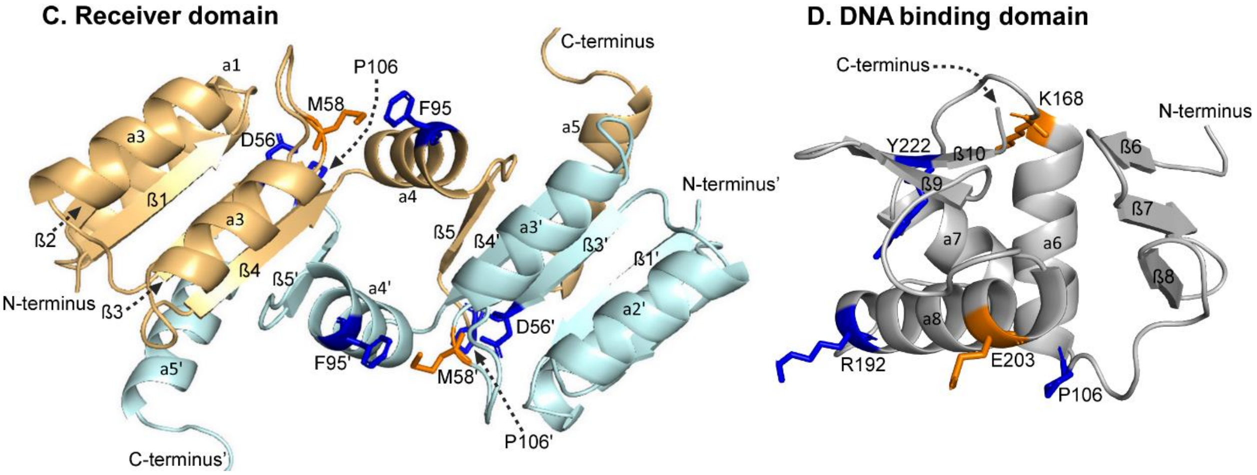


**S2 Fig. The receiver and DNA-binding domains of RR**

(A) Sequence alignment of the receiver domains. The sequences are the following: HitR; BfmR, *Acinetobacter baumannii*; PhoP, *Bacillus subtilis*; YycF, *Bacillus subtilis*; PhoB, *E. coli*; and RstA, *A.*

*baumannii*. (B) Sequence alignment of the DNA-binding domains. The sequences are the following: HitR; PrrA, *Mycobacterium tuberculosis*; PhoB, *E. coli*; SaeR, *Staphylococcus aureus*; VicR, *Streptococcus mutans*; RegX3, *Mycobacterium tuberculosis*; YycF, *Bacillus subtilis*. The α-helices and β-sheets are underlined. The homology modelling was based on HK853 (PDB ID: 4JAU). The homology models of the receiver (C) and DNA-binding domain (D) of HitR were generated based on *M. tuberculosis RegX3* (PDB ID: 2OQR), which is in an active dimer form. Residues identified from genetic selections are highlighted in either orange or blue to specify either ON or OFF mutations, respectively.
