# Supplementary material for: Directed evolution reveals the mechanism of HitRS signaling transduction in Bacillus anthracis
Source: PLoS Pathog. 2020 Dec 23;16(12):e1009148. doi: 10.1371/journal.ppat.1009148 (PMC7790381; doi:10.1371/journal.ppat.1009148)
Supplement: S3 Fig — Growth of B. anthracis WT, WT PhitermC, and isolated activating suppressors in vehicle, erythromycin (20 μg ml-1), or 20 μM ‘205 plus 20 μg ml-1 erythromycin was monitored for 24 h. Data are averages of three independent experiments (mean ± SEM). (PDF) [file ppat.1009148.s006.pdf]

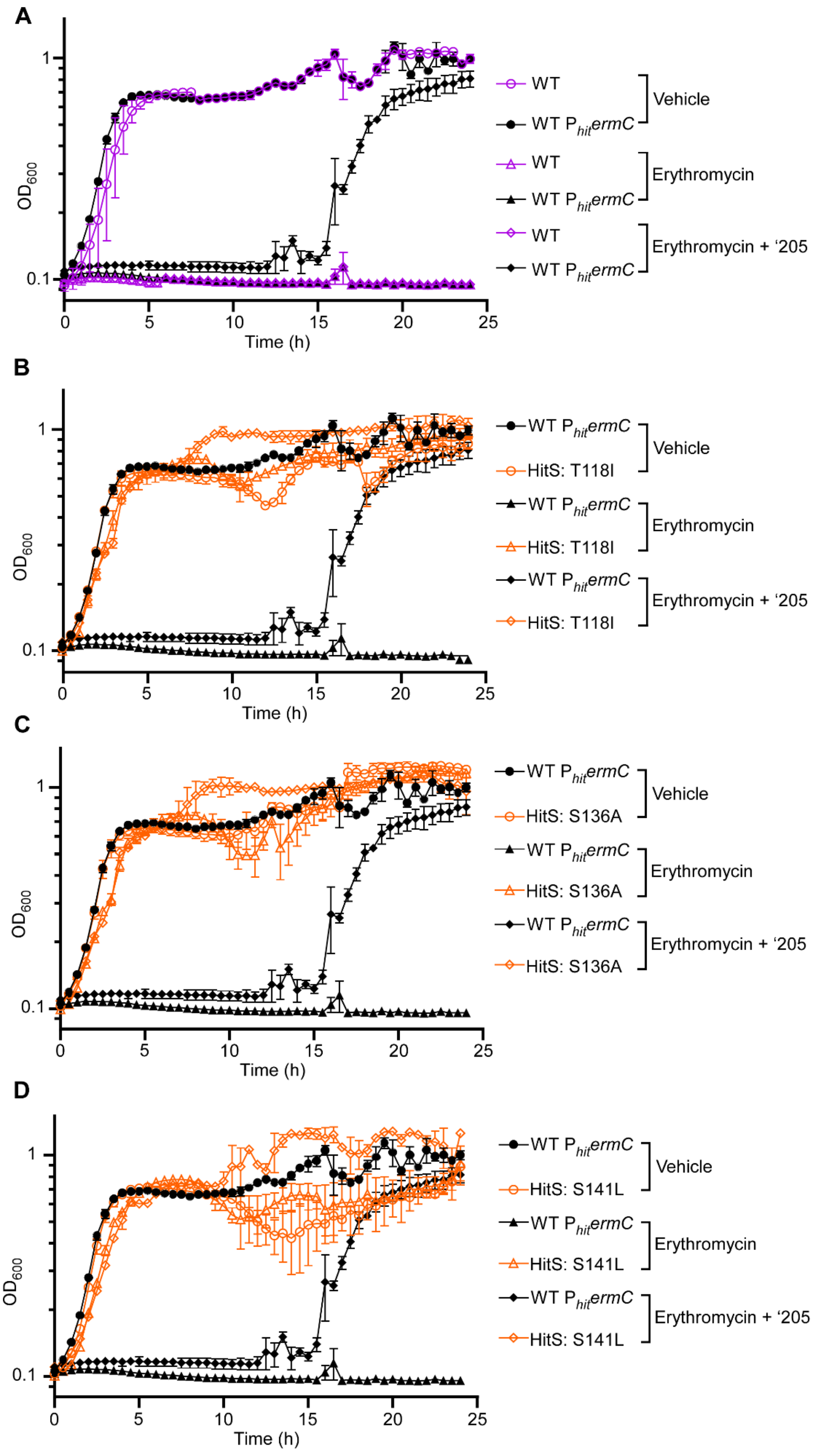
A
OD$_{600}$
1
0.1
0
5
10
15
20
25
Time (h)
WT
WT P_{hit}*ermC*
Vehicle
WT
WT P_{hit}*ermC*
Erythromycin
WT
WT P_{hit}*ermC*
Erythromycin + ‘205
B
OD$_{600}$
1
0.1
0
5
10
15
20
25
Time (h)
WT P_{hit}*ermC*
HitS: T118I
Vehicle
WT P_{hit}*ermC*
HitS: T118I
Erythromycin
WT P_{hit}*ermC*
HitS: T118I
Erythromycin + ‘205
C
OD$_{600}$
1
0.1
0
5
10
15
20
25
Time (h)
WT P_{hit}*ermC*
HitS: S136A
Vehicle
WT P_{hit}*ermC*
HitS: S136A
Erythromycin
WT P_{hit}*ermC*
HitS: S136A
Erythromycin + ‘205
D
OD$_{600}$
1
0.1
0
5
10
15
20
25
Time (h)
WT P_{hit}*ermC*
HitS: S141L
Vehicle
WT P_{hit}*ermC*
HitS: S141L
Erythromycin
WT P_{hit}*ermC*
HitS: S141L
Erythromycin + ‘205

E

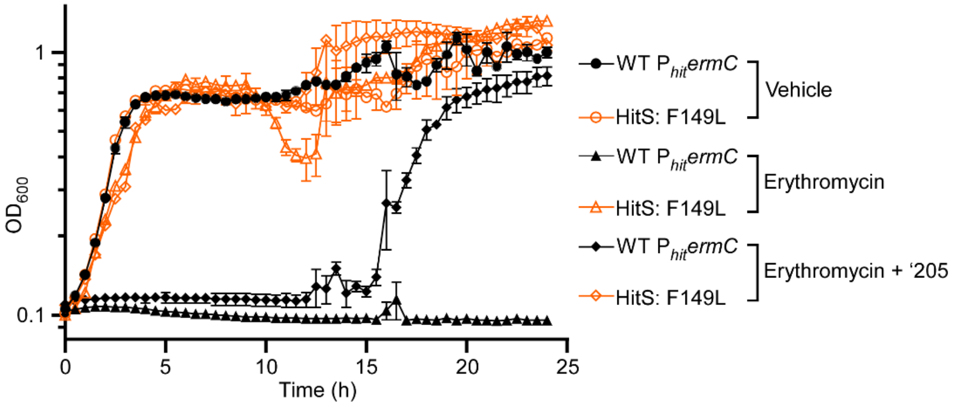

OD600
1
0.1
0
5
10
15
20
25
Time (h)
WT P*hit*ermC
HitS: F149L
Vehicle
WT P*hit*ermC
HitS: F149L
Erythromycin
WT P*hit*ermC
HitS: F149L
Erythromycin + '205

F

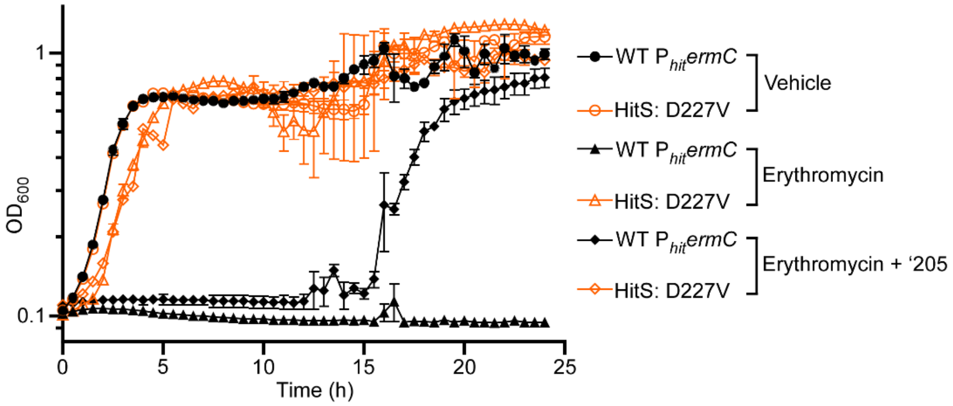

OD600
1
0.1
0
5
10
15
20
25
Time (h)
WT P*hit*ermC
HitS: D227V
Vehicle
WT P*hit*ermC
HitS: D227V
Erythromycin
WT P*hit*ermC
HitS: D227V
Erythromycin + '205

G

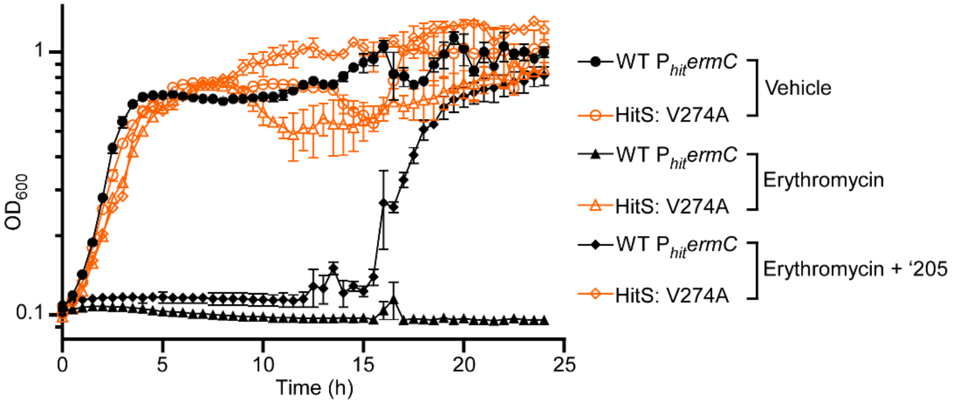

OD600
1
0.1
0
5
10
15
20
25
Time (h)
WT P*hit*ermC
HitS: V274A
Vehicle
WT P*hit*ermC
HitS: V274A
Erythromycin
WT P*hit*ermC
HitS: V274A
Erythromycin + '205

H

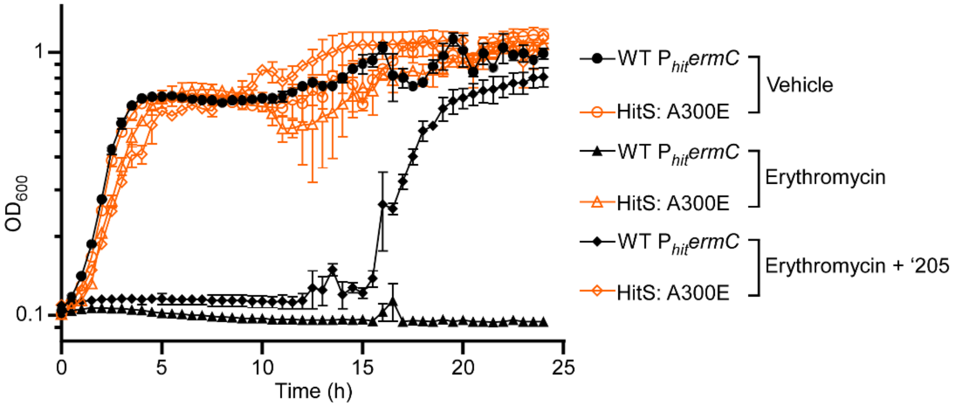

OD600
1
0.1
0
5
10
15
20
25
Time (h)
WT P*hit*ermC
HitS: A300E
Vehicle
WT P*hit*ermC
HitS: A300E
Erythromycin
WT P*hit*ermC
HitS: A300E
Erythromycin + '205

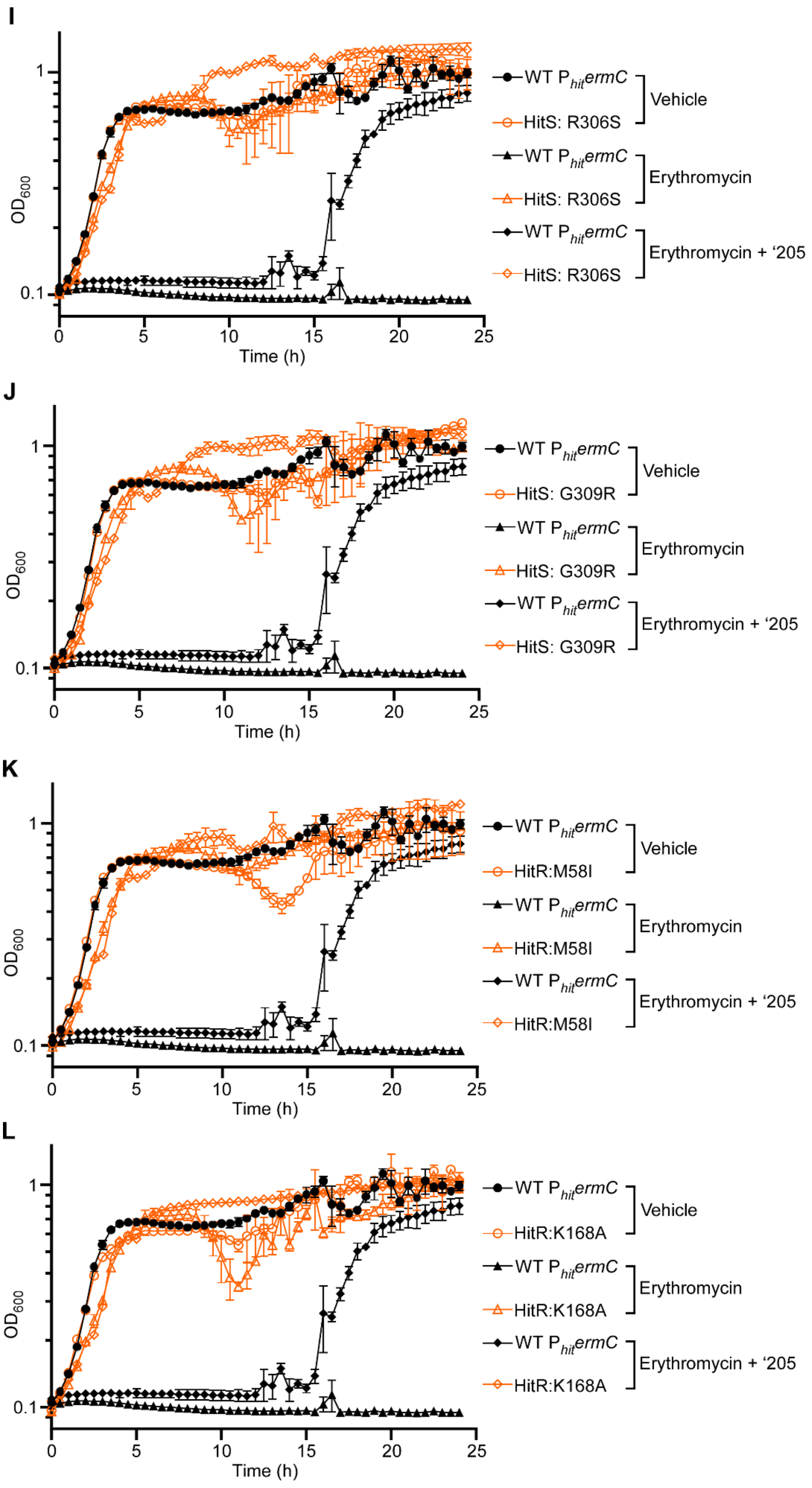
I
OD600
Time (h)
WT P hit ermC
HitS: R306S
Vehicle
WT P hit ermC
HitS: R306S
Erythromycin
WT P hit ermC
HitS: R306S
Erythromycin + ‘205
J
OD600
Time (h)
WT P hit ermC
HitS: G309R
Vehicle
WT P hit ermC
HitS: G309R
Erythromycin
WT P hit ermC
HitS: G309R
Erythromycin + ‘205
K
OD600
Time (h)
WT P hit ermC
HitR:M58I
Vehicle
WT P hit ermC
HitR:M58I
Erythromycin
WT P hit ermC
HitR:M58I
Erythromycin + ‘205
L
OD600
Time (h)
WT P hit ermC
HitR:K168A
Vehicle
WT P hit ermC
HitR:K168A
Erythromycin
WT P hit ermC
HitR:K168A
Erythromycin + ‘205

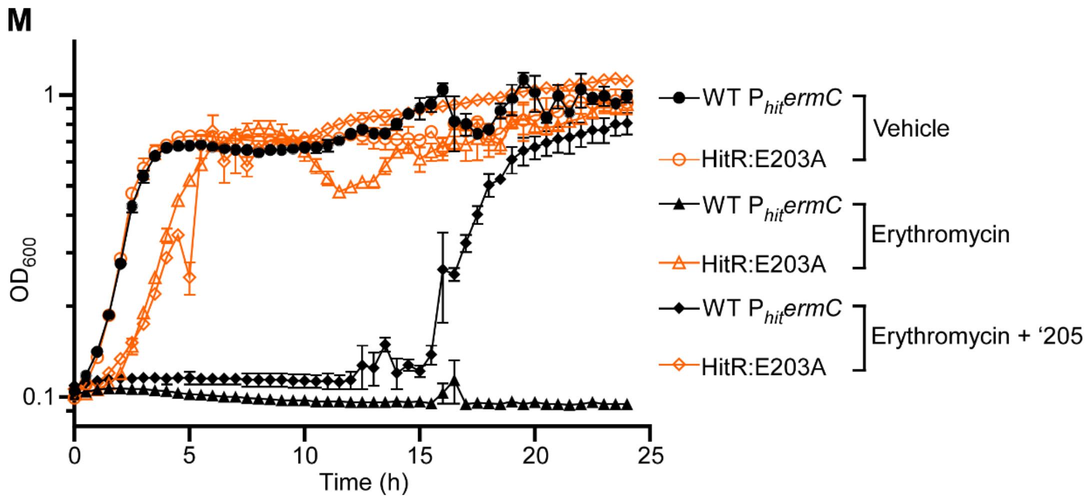

S3 Fig. HitRS point mutants are resistance to erythromycin

Growth of *B. anthracis* WT, WT P$_{hit}$*ermC*, and isolated activating suppressors in vehicle, erythromycin (20 µg ml^{-1}), or 20 µM '205 plus 20 µg ml^{-1} erythromycin was monitored for 24 h. Data are averages of three independent experiments (mean ± SEM).
