# Supplementary material for: Directed evolution reveals the mechanism of HitRS signaling transduction in Bacillus anthracis
Source: PLoS Pathog. 2020 Dec 23;16(12):e1009148. doi: 10.1371/journal.ppat.1009148 (PMC7790381; doi:10.1371/journal.ppat.1009148)
Supplement: S4 Fig — Growth of B. anthracis WT, WT 2(relE) (A-F; WT bas3009::PhitrelE bas4599::PhitrelE), WT 2(relE+ hitRS) (G-N; WT bas3009::PhitrelE bas4599::PhitrelE bas4927::hitRS), and isolated inactivating suppressors. Strains were grown in medium containing vehicle (A, C, E, G, I, K, M) or 20 μM ‘205 (B, D, F, H, J, L, N) and growth was monitored for 24 h. Data are averages of three independent experiments (mean ± SEM). (PDF) [file ppat.1009148.s007.pdf]

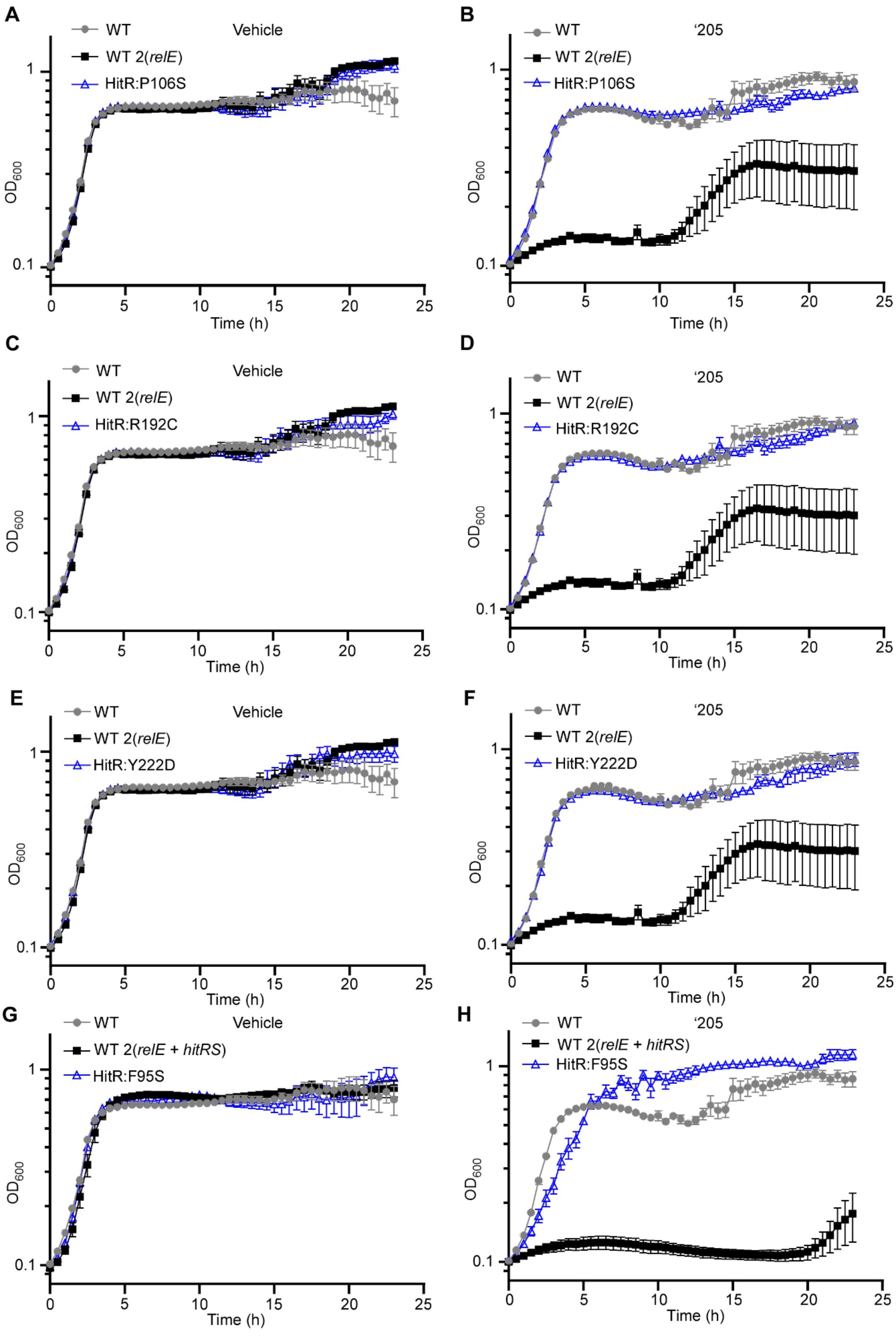

A
WT
Vehicle
WT 2(relE)
HitR:P106S
OD600
Time (h)
B
WT
'205
WT 2(relE)
HitR:P106S
OD600
Time (h)
C
WT
Vehicle
WT 2(relE)
HitR:R192C
OD600
Time (h)
D
WT
'205
WT 2(relE)
HitR:R192C
OD600
Time (h)
E
WT
Vehicle
WT 2(relE)
HitR:Y222D
OD600
Time (h)
F
WT
'205
WT 2(relE)
HitR:Y222D
OD600
Time (h)
G
WT
Vehicle
WT 2(relE + hitRS)
HitR:F95S
OD600
Time (h)
H
WT
'205
WT 2(relE + hitRS)
HitR:F95S
OD600
Time (h)

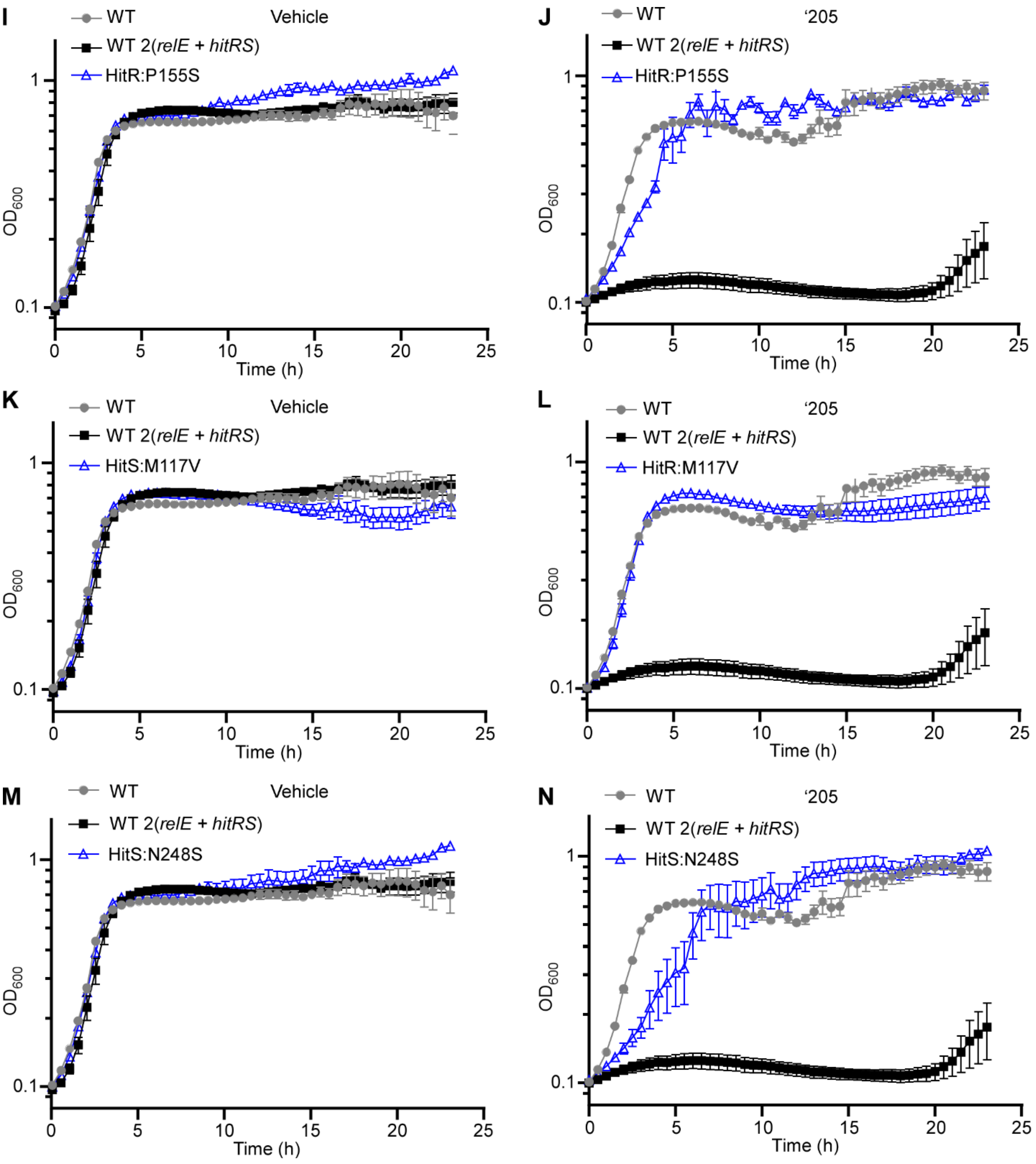

S4 Fig. HitRS point mutants are resistant to '205-mediated killing

Growth of *B. anthracis* WT, WT 2(*relE*) (A-F; WT *bas3009*::P_{hit}*relE bas4599*::P_{hit}*relE*), WT 2(*relE*+ *hitRS*) (G-N; WT *bas3009*::P_{hit}*relE bas4599*::P_{hit}*relE bas4927*::*hitRS*), and isolated inactivating suppressors. Strains

were grown in medium containing vehicle (A, C, E, G, I, K, M) or 20 μM ‘205 (B, D, F, H, J, L, N) and growth was monitored for 24 h. Data are averages of three independent experiments (mean ± SEM).
