# Supplementary material for: Directed evolution reveals the mechanism of HitRS signaling transduction in Bacillus anthracis
Source: PLoS Pathog. 2020 Dec 23;16(12):e1009148. doi: 10.1371/journal.ppat.1009148 (PMC7790381; doi:10.1371/journal.ppat.1009148)
Supplement: S5 Fig — To evaluate the effects of the point mutations on transcription of the hitPRS operon, qPCR was performed. Briefly, overnight cultures were inoculated with a 1:100 ratio into fresh LB medium without (A, C, E; vehicle) or with 20 μM ‘205 (B, D, F). After 6 h of vigorous shaking at 37°C, cells were harvested, and total RNA was extracted and subjected to cDNA synthesis followed by qPCR quantification. All point mutations selected for biochemical characterization were tested: (A-B) constitutively activating and (C-D) inactivating mutants. To confirm the results from the genetic selections using different constructs, representative point mutations were reconstructed in B. anthracis WT background and the effects of these mutations on transcription of the hitPRS operon were tested using qPCR without (E, vehicle) or with 20 μM ‘205 induction (F). The mRNA level of each gene tested in WT untreated cells is set as 1 in both panels. Two sets of oligonucleotide primers were designed to check hitP transcript (hitP1 and hitP2). B. anthracis 16S rRNA was used as a housekeeping control gene. The data are expressed as the mean ± SEM (n = 3). (PDF) [file ppat.1009148.s008.pdf]

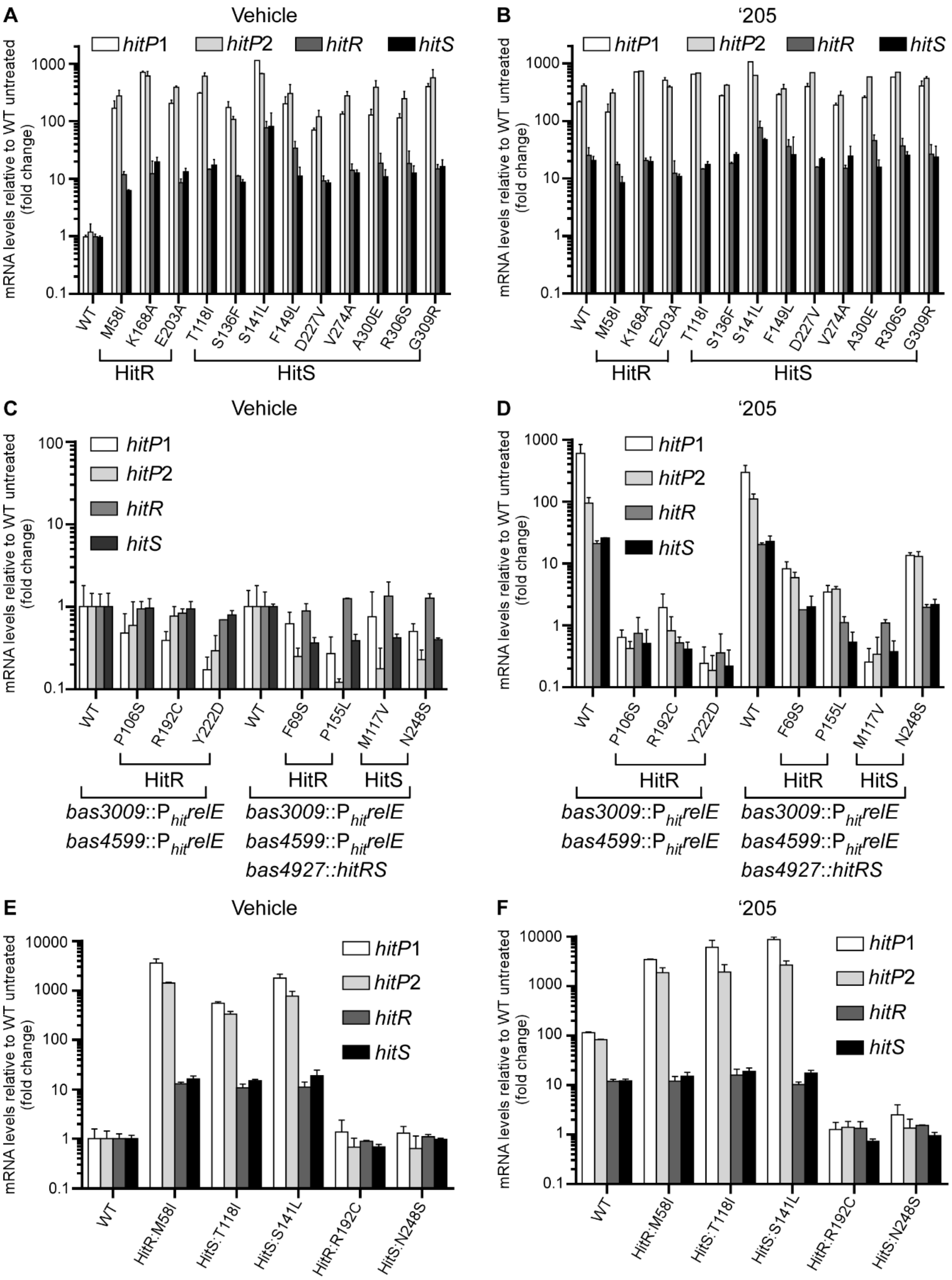

S5 Fig. Point mutations within HitRS affect transcription of *hitPRS*

To evaluate the effects of the point mutations on transcription of the *hitPRS* operon, qPCR was performed. Briefly, overnight cultures were inoculated with a 1:100 ratio into fresh LB medium without (A, C, E; vehicle) or with 20 µM ‘205 (B, D, F). After 6 h of vigorous shaking at 37°C, cells were harvested, and total RNA was extracted and subjected to cDNA synthesis followed by qPCR quantification. All point mutations selected for biochemical characterization were tested: (A-B) constitutively activating and (C-D) inactivating mutants. To confirm the results from the genetic selections using different constructs, representative point mutations were reconstructed in *B. anthracis* WT background and the effects of these mutations on transcription of the *hitPRS* operon were tested using qPCR without (E, vehicle) or with 20 µM ‘205 induction (F). The mRNA level of each gene tested in WT untreated cells is set as 1 in both panels. Two sets of oligonucleotide primers were designed to check *hitP* transcript (*hitP*1 and *hitP*2). *B. anthracis* 16S rRNA was used as a housekeeping control gene. The data are expressed as the mean ± SEM (n=3).
