# Supplementary material for: Directed evolution reveals the mechanism of HitRS signaling transduction in Bacillus anthracis
Source: PLoS Pathog. 2020 Dec 23;16(12):e1009148. doi: 10.1371/journal.ppat.1009148 (PMC7790381; doi:10.1371/journal.ppat.1009148)
Supplement: S6 Fig — To examine the effects of mutations on HitR-DNA-binding, DNA-binding of HitR WT or ON mutants to the target promoter was evaluated using electrophoretic mobility shift assay (EMSA). HitR WT or ON mutants were not subjected to phosphorylation-mediated activation prior to EMSA. The experiments were repeated three times and representative images are shown. (PDF) [file ppat.1009148.s009.pdf]

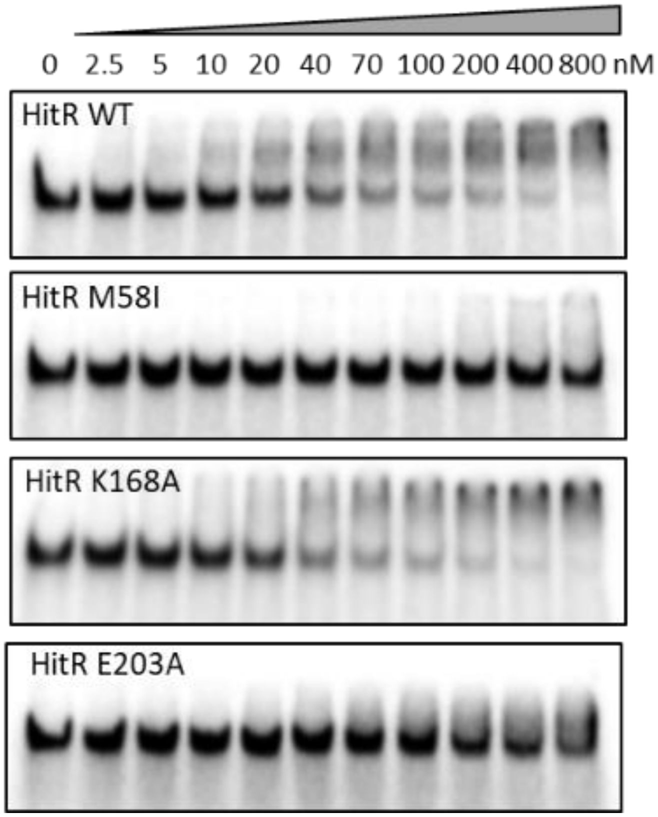

S6 Fig. Phosphorylation-mediated activation is crucial for HitR-DNA-binding

To examine the effects of mutations on HitR-DNA-binding, DNA-binding of HitR WT or ON mutants to the target promoter was evaluated using electrophoretic mobility shift assay (EMSA). HitR WT or ON mutants were not subjected to phosphorylation-mediated activation prior to EMSA. The experiments were repeated three times and representative images are shown.
