# Supplementary material for: Directed evolution reveals the mechanism of HitRS signaling transduction in Bacillus anthracis
Source: PLoS Pathog. 2020 Dec 23;16(12):e1009148. doi: 10.1371/journal.ppat.1009148 (PMC7790381; doi:10.1371/journal.ppat.1009148)
Supplement: S7 Fig — To evaluate the effects of mutation on HitS binding ability to its cognate regulator HitR, a microscale thermophoresis assay was carried out. Briefly, HitR WT protein was labelled with a RED-tris-NTA fluorescence dye at room temperature for 30 min, and 50 nM of the labelled HitR was subsequently incubated with varied concentrations of HitS WT (A) or mutant protein (C and E). In parallel, HitS WT (B) or mutant protein (D and F) was autophosphorylated at 37°C for 30 min before incubation with the labelled HitR. All data points from each independent experiment were plotted and subjected to Kd determination. The Kd values shown are average of three independent experiments (mean ± SEM). (PDF) [file ppat.1009148.s010.pdf]

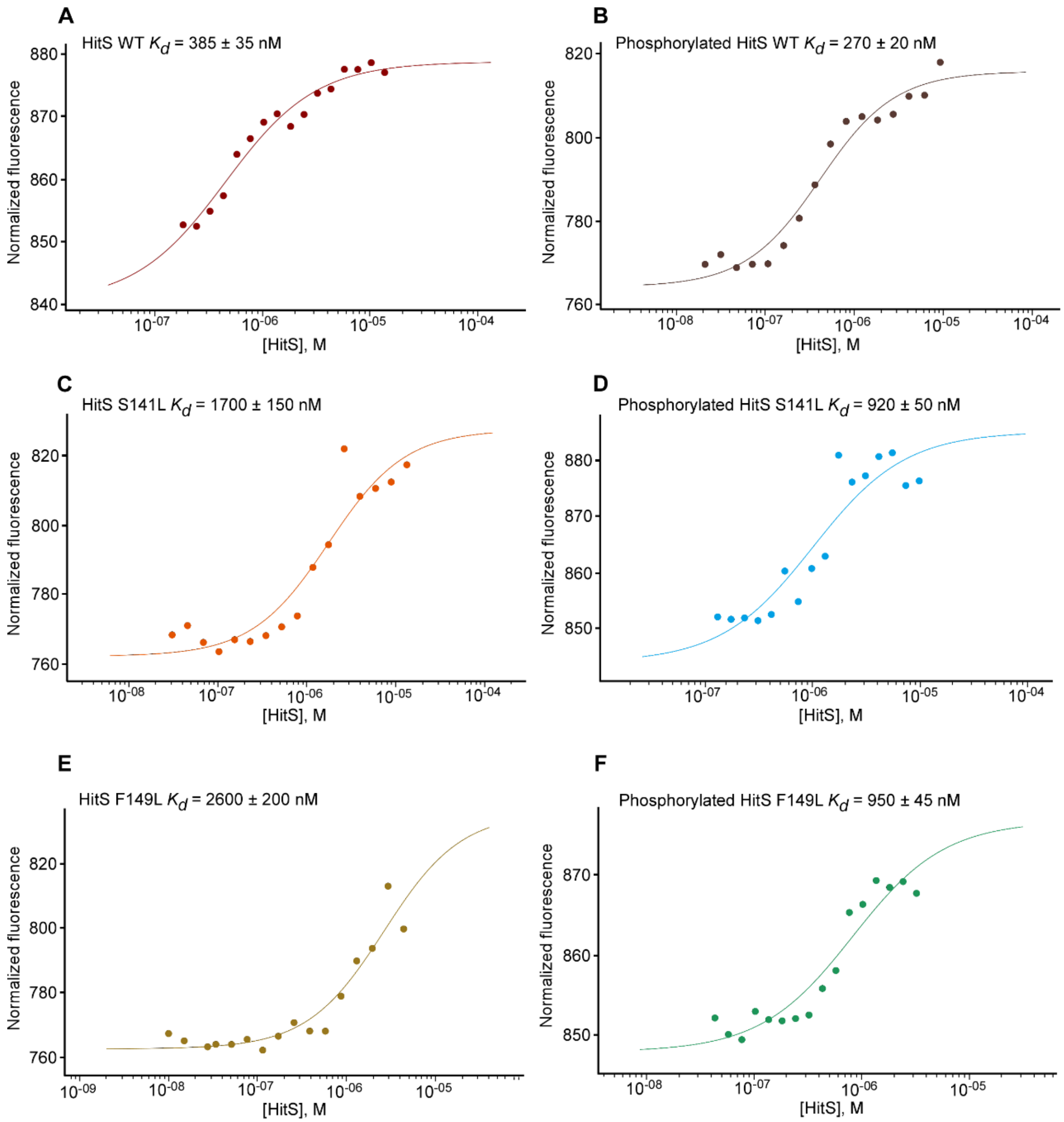

S7 Fig. Residues critical for HitS-HitR interaction

To evaluate the effects of mutation on HitS binding ability to its cognate regulator HitR, a microscale thermophoresis assay was carried out. Briefly, HitR WT protein was labelled with a RED-tris-NTA fluorescence dye at room temperature for 30 min, and 50 nM of the labelled HitR was subsequently incubated with varied concentrations of HitS WT (A) or mutant protein (C and E). In parallel, HitS WT (B)

or mutant protein (D and F) was autophosphorylated at 37°C for 30 min before incubation with the labelled HitR. All data points from each independent experiment were plotted and subjected to K_d determination. The K_d values shown are average of three independent experiments (mean ± SEM).
