# Supplementary material for: Directed evolution reveals the mechanism of HitRS signaling transduction in Bacillus anthracis
Source: PLoS Pathog. 2020 Dec 23;16(12):e1009148. doi: 10.1371/journal.ppat.1009148 (PMC7790381; doi:10.1371/journal.ppat.1009148)
Supplement: Attachment — Submitted filename: Response to Reviewers.pdf [file ppat.1009148.s011.pdf]

RESPONSE TO REVIEWER COMMENTS

REVIEWER 1 COMMENTS

Comment 1: l.87-88: the authors cite compound VU0120205 '205 as a cell-envelope acting compound but the associated reference #23 describes compound VU0038882 ('882) that activates HssRS by inducing endogenous heme biosynthesis in S. aureus. I wonder if the authors could clarify the relationship between HssRS and HitRS. I am not sure how responding to heme availability (HssRS) fits with sensing cell envelope perturbations (HitRS). Also, the authors should explain why they picked HitRS over HssRS.
Response: The reference should be #11 and is now fixed. VU0038882 ('882) activates HssRS but not HitRS. For clarification, we added a sentence in Line 85-86 "These two TCSs cross regulate at both HK-RR and post-RR signaling junctions, suggesting a link between heme toxicity and cell envelope stress."

Comment 2: l. 28: why is Ba referred as an intracellular pathogen?
Response: Anthrax is initiated by Bacillus spores that are phagocytosed by local phagocytes and undergo a transient intracellular stage, which is crucial for its lifecycle. But we agree that most of its lifecycle is extracellular, so we have taken out "intracellular" from the text in Line 28.

Comment 3: l. 36: this is a very strong statement. I don't think that the study comes close to laying such a foundation. The "Author Summary" section uses slightly different words but essentially repeats the abstract.
Response: Based on our unpublished data from another study, the HitRS system is activated during phagocytosis and confers a fitness advantage to bacterial survival within phagocytes. Therefore, this system could be a potential target for antimicrobial therapeutics. We think this study with detailed structure-function analysis could be the first step to achieve the goal. Furthermore, these signaling proteins (HKs and RRs) are well conserved, the ON and OFF mutations identified from this study could serve as a comprehensive map for other TCSs with unknown targets/partners in other pathogens. However, we agree with the referee's comments and have taken out the statement from the Abstract.

Comment 4: l.49: it is odd to start the paper with a statement about the rising incidence of antibiotic resistance. That is not a trait associated with Ba. Word on the street is that this pathogen is dormant in soils; mostly! Certainly, Ba isn't roaming around hospitals with the Enterococci and the likes of them.
Response: We agree with the referee's comments and took out the text regarding rising incidence of antibiotic resistance from the text and rephrase the first paragraph of the Introduction section (Line 50-59).

Comment 5: l.58: during infection Ba is only transiently intracellular.
Response: As described above, we have taken out "intracellular" from the text in Line 58.

Comment 6: Fig. 1 and corresponding Result section present clever approaches to isolate hit alleles with constitutive activity and mutations that inactivate signaling or signal transduction. However, this does not bring the authors closer to identifying the signal that activates HitRS. As expected mutations arising from these selections bypass the requirement for the true ligand of HitRS.
Response: We agree with the referee's comments. Indeed, Fig. 1 and corresponding Result section only allow us to identify residues critical for HitRS signal transduction. The identification of the activating signal is beyond the scope of this study and is actually another ongoing project in the lab.

REVIEWER 2 COMMENTS

Comment 1. Fig. S3. This schematic represents a major set of results of this study. Consider moving it to the main text (perhaps in Fig. 1?).
Response: We agree with the referee's comments and moved Fig. S3 to Fig.1.
